# Androgen deprivation therapy improves the in vitro capacity of high-density lipoprotein (HDL) to receive cholesterol and other lipids in patients with prostate carcinoma

**DOI:** 10.1186/s12944-020-01305-8

**Published:** 2020-06-10

**Authors:** Cicero P. Albuquerque, Fatima R. Freitas, Ana Elisa M. Martinelli, Josefa H. Lima, Rafael F. Coelho, Carlos V. Serrano Jr., Willian C. Nahas, Roberto Kalil Filho, Raul C. Maranhão

**Affiliations:** 1 Instituto de Coracao, Hospital das Clinicas HCFMUSP, Faculdade de Medicina, Universidade de Sao Paulo, Sao Paulo, Brazil; 2 Instituto do Cancer do Estado de São Paulo, Hospital das Clinicas HCFMUSP, Faculdade de Medicina, Universidade de São Paulo, São Paulo, Brazil; 3 Faculdade de Ciencias Farmaceuticas, Universidade de Sao Paulo, Sao Paulo, Brazil

**Keywords:** Androgen deprivation therapy (ADT), Prostate cancer, Lipid metabolism, Cholesterol transfer, High-density lipoprotein (HDL)

## Abstract

**Background:**

Androgen deprivation therapy (ADT) is widely used in the treatment of testosterone-dependent prostate carcinomas. ADT often increases plasma LDL and HDL cholesterol and triglycerides. The aim was to test whether ADT changes the transfer of lipids to HDL, an important aspect of this metabolism and HDL protective functions, and related parameters.

**Methods:**

Sixteen volunteers with advanced prostate carcinoma submitted to pharmacological ADT or orchiectomy had plasma collected shortly before and after 6 months of ADT. In vitro transfer of lipids to HDL was performed by incubating plasma with donor emulsion containing radioactive lipids by 1 h at 37 °C. After chemical precipitation of apolipoprotein B-containing lipoprotein, the radioactivity of HDL fraction was counted.

**Results:**

ADT reduced testosterone to nearly undetectable levels and markedly diminished PSA. ADT increased the body weight but glycemia, triglycerides, LDL and HDL cholesterol, HDL lipid composition and CETP concentration were unchanged. However, ADT increased the plasma unesterified cholesterol concentration (48 ± 12 vs 56 ± 12 mg/dL, *p* = 0.019) and LCAT concentration (7.15 ± 1.81 vs 8.01 ± 1.55μg/mL, *p* = 0.020). Transfer of unesterified (7.32 ± 1.09 vs 8.18 ± 1.52%, *p* < 0.05) and esterified cholesterol (6.15 ± 0.69 vs 6.94 ± 1.29%, *p* < 0.01) and of triglycerides (6.37 ± 0.43 vs 7.18 ± 0.91%, *p* < 0.001) to HDL were increased after ADT. Phospholipid transfer was unchanged.

**Conclusion:**

Increase in transfer of unesterified and esterified cholesterol protects against cardiovascular disease, as shown previously, and increased LCAT favors cholesterol esterification and facilitates the reverse cholesterol transport. Thus, our results suggest that ADT may offer anti-atherosclerosis protection by improving HDL functional properties. This could counteract, at least partially, the eventual worse effects on plasma lipids.

## Introduction

Androgen deprivation therapy (ADT), as achieved by both pharmacological and surgical castration, is an efficient and widely used therapeutic tool for the control of testosterone-dependent prostate carcinomas. ADT is often adopted in relapsing tumors after radical prostatectomy or radiotherapy [1, 2]. The possible metabolic consequences of ADT, such as bone loss and development of metabolic syndrome, a condition that predisposes to cardiovascular disease, are major concerns in the follow-up of the patients [1, 3].

Together with glucose intolerance, systemic arterial hypertension, overweight and high plasma triglycerides, low high-density lipoprotein (HDL)-cholesterol is one of the cardinal components of metabolic syndrome. HDL is considered a major anti-atherogenic defense of the organism since HDL-cholesterol levels are inversely correlated with the incidence of atherosclerotic cardiovascular diseases [4, 5].

HDL receives cholesterol from the cells of the peripheral tissues for excretion by the liver in the bile, in the so-called reverse cholesterol transport. Cholesterol from other lipoproteins and from the cells transferred to HDL is esterified in this lipoprotein fraction by lecithin cholesterol acyl transferase (LCAT) using apolipoprotein (apo) A-I, the main HDL apo, as co-factor [6]. Esterification of cholesterol stabilizes the cholesterol plasma pool and drives the reverse cholesterol transport, so that the rates of transfer of cholesterol to HDL may be important to HDL functional role [6]. HDL has also several protective actions, such as those of anti-oxidation, vasodilation, anti-inflammatory, anti-apoptotic, anti-thrombotic, anti-infectious actions and is a major transporter of microRNA’s that regulates several metabolic processes [4, 6]. In this setting, the HDL-cholesterol levels do not predict the full protective role of the lipoprotein and functional tests of the lipoprotein have been developed.

This study was aimed to investigate in patients with prostate carcinoma whether ADT may change the transfers of cholesterol and other lipids to HDL. By means of an in vitro assay, the simultaneous transfer to HDL of unesterified and esterified cholesterol, phospholipids and triglycerides from a donor artificial lipid emulsion labeled with radioactive lipids was measured [7] in patients undergoing either pharmacological or surgical ADT. The effects of ADT on the classical plasma lipid parameters and on cholesteryl ester transfer protein (CETP), that facilitates lipid transfers, were also documented.

## Methods

### Study subjects

Sixteen volunteers man, aged between 60 and 80 years (72 ± 7 yrs), with advanced or metastatic prostate cancer, which had been scheduled for surgical (7 patients) or pharmacological (9 patients) testosterone depletion as treatment of the disease, were selected for the study. ADT was defined as administration of GnRH (gonadotropin-releasing hormone) agonist, goserelin acetate (Zoladex, AstraZeneca, London, UK), 10.8 mg every 3 months, or total orchiectomy. Before and after 6 months of ADT, the blood of the patients was collected to analyses.

The exclusion criteria were: previously received treatments for prostate cancer, concomitant therapy during the study, and manifested cardiovascular or metabolic diseases. Patients did not change their lifestyle during ADT.

The study was approved by the Ethics Committee of the University of São Paulo. All subjects gave written informed consent in accordance with the Declaration of Helsinki.

### Biochemical determination

The blood samples were collected after 12-h fasting. Testosterone, prostate-specific antigens (PSA), glucose, triglycerides and total cholesterol and HDL-cholesterol were determined by commercial enzymatic colorimetric methods (Dimension RXL, Siemens Healthcare, Newark, NJ, USA). Plasmatic unesterified cholesterol was also determined by an enzymatic colorimetric method (Wako, Richmond, VA, USA). Low-density lipoprotein (LDL)-cholesterol was calculated by the Friedewald eq**.** [8] and non-HDL-cholesterol was determined by the equation: total cholesterol minus HDL-cholesterol.

### Lipid composition of HDL fraction

The HDL fraction was obtained from the whole plasma after precipitation of the apolipoprotein B-containing lipoproteins with magnesium phosphotungstate. Triglyceride, total cholesterol, phospholipid (Labtest, Vista Alegre, Brazil), and unesterified cholesterol (Wako) were determined by using commercial kits. Esterified cholesterol was calculated as the difference between total and unesterified cholesterol multiplied by 1.67 to adjust molecular weight [9].

### Determination of CETP and LCAT concentration

Plasmatic concentrations of CETP and LCAT were determined by immunoassay (ALPCO Diagnostics, Salem, NH, USA).

### Preparation of the artificial lipid nanoparticle

The radioactively labeled lipid donor nanoparticle was prepared from a lipid mixture described previously by Ginsburg et al. [10] and modified by Maranhão et al. [11]. Two sets of the nanoemulsion were prepared, one labeled with 3H-triglycerides and 14C-cholesterol and the other with 3H-cholesteryl esters and 14C-phospholipids. In a vial, 40 mg cholesteryl oleate, 20 mg egg phosphatidylcholine, 1 mg triolein and 0.5 mg cholesterol, purchased from Sigma Aldrich (St. Louis, MO, USA), were mixed. Trace amounts of glycerol tri [[9, 10](n)-^3^H] oleate and 4-^14^C-cholesterol or [1α,2α(n)-^3^H]-cholesteryl oleate and L-3-phosphatidylcholine,1-stearoyl-2-[1-^14^C] arachidonyl (Amersham BioSciences, Little Chalfont, Buckinghamshire, UK) were added to the initial solution. The lipids were emulsified by prolonged ultrasonic irradiation in aqueous media for 3 h, and the crude emulsion then ultracentrifugated in a two-step process, with density adjustment by addition of KBr to obtain the nanoparticle. The nanoparticle fraction was dialyzed against a 0.9% NaCl solution.

### Lipid transfer from the donor nanoparticle to HDL

The in vitro assay of lipid transfer from the lipid nanoparticle to HDL was previously described by Lo Prete et al. [7]. An aliquot of 200 μL of the plasma with EDTA was incubated with 50 μL of the nanoparticle labeled with ^14^C-cholesterol and ^3^H-triglycerides or with ^3^H-cholesteryl esters and ^14^C-phospholipids, under agitation, during 1 h at 37 °C. After incubation, 250 μL of dextran sulfate/MgCl_2_ was added as precipitation reagent for the apolipoprotein B-containing lipoproteins. The mixture was shaken for 30 s, centrifuged for 10 min at 3000 g. Aliquots of 250 μL of the supernatant, containing by HDL fraction, were added in 5 mL of scintillation solution (Packard BioScience, Groeningen, Netherlands) and the radioactivity was measured in liquid scintillation analyzer (Packard BioScience). Radioactivity was then measured with Packard 1600 TR model Liquid Scintillation Analyzer (Packard BioScience). The transfer of each lipid from the nanoparticle to the HDL fraction was expressed as % of the total incubated radioactivity, determined in a plasma sample without the addition of precipitation reagent. The radioactivity in the precipitate, which comprised the donor emulsion and the apo B-containing lipoproteins such as VLDL and LDL, was not counted and the precipitate was discarded.

Changes in pH and addition of albumin to the incubates did not influence the results of the in vitro transfer assay, as previously tested [7]. To verify the purity of HDL after the incubation with the donor emulsion and of the chemical precipitation procedure, apo B and apo A-I were determined in the supernatant fraction. In all experiments, apo B was always absent from the supernatant, indicating that it was not contaminated by lipoprotein classes other than HDL. In presence of the emulsion, the supernatant apo A-I diminished, which is predictable since apo B containing lipoproteins also contain apo A-I, the main HDL apolipoprotein. The % HDL lipid composition, on the other hand, did not substantially change after incubation and precipitation in the presence or not of the donor emulsion. As measured by the dynamic laser scattering, the diameter of the HDL particles in the supernatant was not changed after incubation with the donor emulsion.

### Statistical analyses

Statistical analyzes were conducted using SPSS 19.0 statistical software (SPSS® Advanced Statistics, IBM Corporation, Illinois, USA). Shapiro-Wilk test was performed to evaluate Gaussian distribution. Data were compared using the paired t test for Gaussian distribution data and the Wilcoxon test for non-Gaussian distribution data. The results are expressed as mean ± SD. In all analyses, parameters were considered significantly different when *p* < 0.05.

## Results

As shown in Table 1, the testosterone serum concentration was lowered to < 12 ng/dL after ADT. In all patients, ADT was capable of efficiently reducing the prostate cancer marker PSA (%Δ = -84.7; *p* = 0.001). The plasma glucose levels were unaffected by the treatments. The plasma lipid parameters, namely total, LDL, non-HDL and HDL cholesterol, as well as triglycerides were also unchanged after ADT (Table 2). The total concentration in the plasma of unesterified cholesterol was, however, increased after ADT. Likewise, ADT did not change the lipid composition of the HDL fraction. LCAT concentration, but not CETP, was increased after the ADT (*p* < 0.020).

**Table 1:** Physical characteristics of patients with prostate cancer before and after androgen deprivation therapy (*n* = 16)

	Basal	Post-treatment	*P*-value
Weight (kg)	71.0 ± 13.1	74.5 ± 13.9	***0.0001***
BMI (kg/m^2^)	26.7 ± 4.9	27.0 ± 3.4	0.660
Testosterone (ng/dL)	527 ± 226	< 12	***0.001***
PSA (ng/mL)	361 ± 508	13 ± 21	***0.001***

*BMI* Body mass index, *PSA* Prostate-specific antigens

**Table 2:** Serum biochemistry data and HDL composition of patients with prostate cancer before and after androgen deprivation therapy (*n* = 16)

	Basal	Post-treatment	*P*-value
Glycemia (mg/dL)	103 ± 16	102 ± 17	0.909
Cholesterol (mg/dL)			
total	194 ± 47	209 ± 37	0.112
LDL	119 ± 43	133 ± 30	0.194
HDL	48 ± 11	51 ± 15	0.191
non-HDL	146 ± 46	158 ± 34	0.159
unesterified	48 ± 12	56 ± 12	***0.019***
Triglycerides	111 ± 54	127 ± 55	0.079
LCAT (μg/mL)	7.15 ± 1.81	8.01 ± 1.55	***0.020***
CETP (μg/mL)	2.47 ± 0.78	2.48 ± 0.77	0.999
HDL lipid composition (%)			
Esterified cholesterol	43.2 ± 8.8	43.5 ± 11.0	0.880
Unesterified cholesterol	6.4 ± 2.5	6.9 ± 2.4	0.352
Triglycerides	9.5 ± 3.2	9.6 ± 3.0	0.923
Phospholipids	49.8 ± 25.1	39.1 ± 26.6	0.073

*LDL* Low density lipoprotein, *HDL* High density lipoprotein, *LCAT* lecithin cholesterol acyl transferase, *CETP* Cholesteryl ester transfer protein

**Fig. 1:**
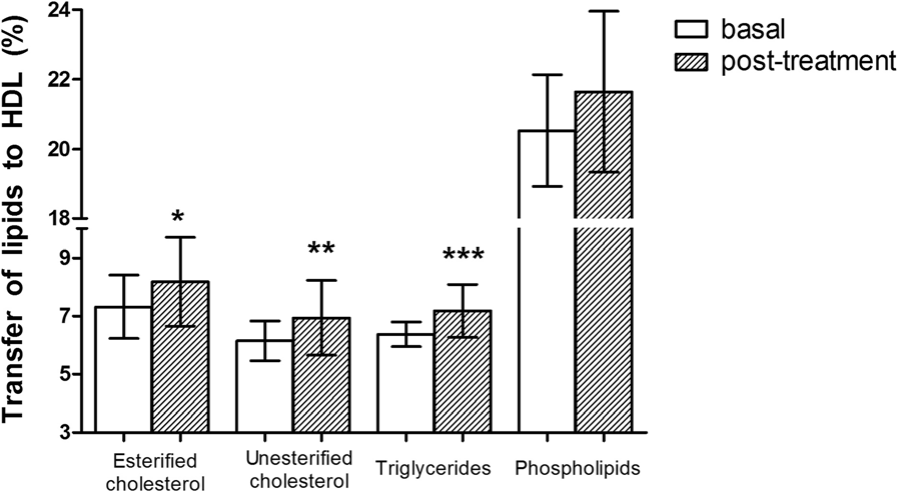
In vitro transfers of unesterified and esterified cholesterol, triglycerides and phospholipids from the donor lipid nanoemulsion to HDL (high density lipoprotein) of patients with prostate cancer before and after androgen deprivation therapy (*n* = 16). * *p* < 0.05, ** *p* < 0.01, *** *p* < 0.001 post-treatment vs basal

Fig. 1 shows the effects of the treatments on the transfer of the four radioactive lipids from the donor lipid emulsion to the HDL fraction. The transfer of unesterified (from 7.32 ± 1.09 to 8.18 ± 1.52%, *p* < 0.05) and esterified cholesterol (from 6.15 ± 0.69 to 6.94 ± 1.29%, *p* < 0.01) and of triglycerides (from 6.37 ± 0.43 to 7.18 ± 0.91%, *p* < 0.001) were increased after the treatments. The trend to increase the phospholipid transfer was not statistically significant (from 20.53 ± 1.60 to 21.65 ± 2.31%, *p* = 0.075).

When comparing the data of the nine patients treated with goserelin with the seven that underwent surgical castration, there was no statistically difference between the two groups regarding all the measured parameters (data not shown).

## Discussion

In this study, in which the plasma testosterone was reduced to nearly undetectable levels 6 months after the commencement of ADT, statically significant changes in the plasma concentration of glucose, triglycerides and LDL-cholesterol and non-HDL-cholesterol and HDL-cholesterol were not observed.

It is generally agreed that ADT increases insulin resistance [12–14]. In some studies, glycemia was indeed increased [15, 16] but in others it was unchanged [12, 17], as we found here. In the study by Saglam et al. [18], glycemia increased only after 6-month ADT. Heterogeneous results were found regarding the plasma lipid fractions: increased or unaltered LDL-cholesterol or triglycerides have been reported [3, 12, 16, 19, 20]. Some authors found unaltered HDL-cholesterol but either reduction or increase in this fraction were also observed [3, 12, 15, 16, 20, 21]. ADT increases body weight [3, 19, 22], as also occurred in our patients. Overweight and obesity may raise glycemia, triglyceridemia and LDL-cholesterol and may eventually account for the alterations in plasma lipids by ADT found in some studies.

Lipid transfers from the other lipoprotein classes to HDL, tested here by the in vitro assay using the artificial emulsion as lipid donor, depend on several factors. The action of the transfer proteins, CETP and the phospholipid transfer protein (PLTP), that facilitate the shift of lipid species among the lipoproteins is of great importance. The concentration in the plasma of the apo B-containing lipoproteins that will compete with HDL for receiving the lipids and the concentration and the composition of HDL itself can also account for the overall results of the assay. In this in vitro assay, most of the factors that may affect the in vivo lipid transfers to HDL are present in the incubated plasma, such as the transfer proteins, LCAT and apo A-I, apo B-containing lipoproteins and so forth [6, 7].

After ADT, there was an increase in the in vitro transfer to HDL of three of the four lipids measured here, namely esterified and non-esterified cholesterol and triglycerides. This occurred despite the lack of increase in the HDL-cholesterol levels. Indeed, HDL-cholesterol is only one of the factors that can influence the lipid transfer rates that are consistently independent parameters [6].

In previous studies, it was shown that the transfer of unesterified cholesterol to HDL was diminished in patients with precocious coronary artery disease (CAD) [23]. Similarly, in patients with CAD and type 2 diabetes mellitus unesterified cholesterol transfer was also diminished compared with diabetes without CAD and, in addition, the esterified cholesterol transfers were reduced [24]. Moreover, in acute myocardial infarction and in conditions that predispose to CAD manifestations, such as sedentarism, rheumatoid arthritis, patients with heart graft or myocardial infarction and bedridden patients, the cholesterol transfer to HDL is reduced ([25–28], see ref. [6] for review). On the other hand, conditions that are favorable to atherosclerosis prevention, such as physical training, increase the transfers of cholesterol to HDL [29]. Therefore, the results of the previous studies suggest that the presence of cardiovascular disease or other unhealthy conditions are associated with decrease in the transfer of cholesterol to the HDL fraction. It can be assumed that the cholesterol amounts that are not transferred to HDL would tend to remain in the atherogenic apo B-containing lipoprotein fractions.

In this study, 6 months after ADT did not develop the metabolic syndrome, features that sometimes has been reported in patients under ADT [3]. In this regard, Casella-Filho et al. [30] showed that metabolic syndrome patients had diminished transfers of both unesterified and esterified cholesterol, which were reversed by physical training. These findings highlight the importance of preventive measures, such as physical training and weight loss in ADT patients against the development of insulin resistance and metabolic syndrome. In subjects with type 2 diabetes without CAD, the glucose levels did not determine changes in the lipid transfers [31].

In this study, CETP concentration in the plasma did not change after ADT. It is worthwhile to point out that while the exchange among lipoproteins of esterified cholesterol, triglycerides are largely dependent on CETP, the transfer of unesterified cholesterol is spontaneous and not influenced by actions of this protein [32].

Interestingly, the LCAT concentration increased but the unesterified/total cholesterol ratio in whole plasma did not decrease. This finding can be ascribed to the compartmental behavior and exchanges of cholesterol in the plasma and tissues [33, 34].

In the study by Østergren et al. [35], in regard to our results, it is of note to point out that surgical and pharmacological ADT were not different in respect to all measured parameters. However, comparisons of the effects of GnRH agonist and orquiectomy in the risk of CVD are controversial [36–38]. In the present study, the number of studied patients in each group is probably insufficient for reliable conclusions.

It is debatable whether or not HDL might offer protective effects against the genesis and progression of different cancer types [39, 40], including prostate carcinoma, but exploring HDL functions in cancer is a tempting avenue of research. In this respect, investigation of the cholesterol efflux from cells to HDL, the first step of cholesterol reverse transport would add an interesting piece of information on the effects of hormonal therapy. In a recent study, we showed that in patients with acute myocardial infarction, there was convergence of data from the in vitro lipid transfer assay and the data from cholesterol efflux from J774 macrophages. In the myocardial infarction patients both transfer of cholesterol from the donor emulsion to HDL and the cholesterol efflux from macrophages to HDL were diminished [28]. Overall, it is rather debatable whether or not ADT administered to patients with prostate carcinoma might lead to increase in the incidence of atherosclerotic cardiovascular disease.

The small number of patients can be mentioned as limitation of this study to be considered in the design of future studies exploring hormonal influences on HDL metabolism and function.

## Conclusion

The current finding that ADT increases the transfer of cholesterol to HDL, an important aspect of this metabolism, suggests that ADT may also offer protective actions that could help to counteract the burdens of eventual adverse change in this metabolism. Our results encourage the exploration of other functional aspects of HDL under ADT, such as the anti-oxidant and the anti-inflammatory actions of this lipoprotein.

## Data Availability

The datasets generated during and/or analyzed during the current study are available from the corresponding author on reasonable request.

## Abbreviations

ADT: Androgen deprivation therapy
apo: Apolipoprotein
CAD: Coronary artery disease
CETP: Cholesteryl ester transfer protein
GnRH: Gonadotropin-releasing hormone
HDL: High-density lipoprotein
LCAT: Lecithin cholesterol acyl transferase
LDL: Low-density lipoprotein
PLTP: Phospholipid transfer protein
PSA: Prostate-specific antigens
